# Identification of miRNA and Their Regulatory Effects Induced by Total Flavonoids From *Dracocephalum moldavica* in the Treatment of Vascular Dementia

**DOI:** 10.3389/fphar.2021.796628

**Published:** 2021-12-06

**Authors:** Mimin Liu, Guangzhi Shan, Hailun Jiang, Li Zeng, Kaiyue Zhao, Yiran Li, Ghulam Md Ashraf, Zhuorong Li, Rui Liu

**Affiliations:** ^1^ Institute of Medicinal Biotechnology, Chinese Academy of Medical Sciences and Peking Union Medical College, Beijing, China; ^2^ Pre-Clinical Research Unit, King Fahd Medical Research Center, King Abdulaziz University, Jeddah, Saudi Arabia; ^3^ Department of Medical Laboratory Technology, Faculty of Applied Medical Sciences, King Abdulaziz University, Jeddah, Saudi Arabia

**Keywords:** bioinformatics, *Dracocephalum moldavica* L., flavonoids, microRNA, network regulation, vascular dementia

## Abstract

Vascular dementia (VaD) is a general term used to describe difficulties in memory, reasoning, judgment, and planning caused by a reduced blood flow to the brain and consequent brain damage, in which microRNAs (miRNAs) are involved. *Dracocephalum moldavica* L. (*D. moldavica*) is traditionally used in the treatment of cardiovascular diseases as well as VaD, but the biomolecular mechanisms underlying its therapeutic effect are obscure. In the present study, the molecular mechanisms involved in the treatment of VaD by the total flavonoids from *Dracocephalum moldavica* L. (TFDM) were explored by the identification of miRNA profiling using bioinformatics analysis and experimental verification. A total of 2,562 differentially expressed miRNAs (DEMs) and 3,522 differentially expressed genes (DEGs) were obtained from the GSE120584 and GSE122063 datasets, in which the gene functional enrichment and protein-protein interaction network of 93 core targets, originated from the intersection of the top DEM target genes and DEGs, were established for VaD gene profiling. One hundred and eighty-five targets interacting with 42 flavonoids in the TFDM were included in a compound-target network, subsequently found that they overlapped with potential targets for VaD. These 43 targets could be considered in the treatment of VaD by TFDM, and included CaMKII, MAPK, MAPT, PI3K, and KDR, closely associated with the vascular protective effect of TFDM, as well as anti-oxidative, anti-inflammatory, and anti-apoptotic properties. The subsequent analysis of the compound-target gene-miRNA network indicated that eight miRNAs that mediated 43 targets had a close interaction with TFDM, suggesting that the neuroprotective effects were principally due to kaempferol, apigenin, luteolin, and quercetin, which were mostly associated with the miR-3184-3p/ESR1, miR-6762-3p/CDK1, miR-6777-3p/ESRRA, and other related axes. Furthermore, the *in vitro* oxygen-glucose deprivation (OGD) model demonstrated that the dysregulation of miR-3184-3p and miR-6875-5p found by qRT-PCR was consistent with the changes in the bioinformatics analysis. TFDM and its active compounds involving tilianin, luteolin, and apigenin showed significant effects on the upregulation of miR-3184-3p and downregulation of miR-6875-5p in OGD-injured cells, in line with the improved cell viability. In conclusion, our findings revealed the underlying miRNA-target gene network and potential targets of TFDM in the treatment of VaD.

## Introduction

Vascular dementia (VaD) represents a group of syndromes characterized by cognitive impairment resulting from the death of the hypoxic brain tissue caused by the reduced perfusion from diseased brain blood vessels (Iadecola, 2013), causing approximately 15–20% of all dementia cases (O'Brien and Thomas, 2015). The pathogenesis of VaD is complex and is the result of a variety of cardiovascular or genetic risk factors associated with Apolipoprotein E (APOE), angiotensin I converting enzyme (ACE), paraoxonase 1 (PON1), or presenilin 1 (PSEN1) genes, or due to stroke (O’Brien and Thomas, 2015). Thus, there is no authorized treatment for VaD in clinical practice. The current therapies for VaD are mostly performed using cholinesterase inhibitors or memantine due to the lack of a reliable biomarker or an established molecular mechanism, and these compounds are moderately effective. However, among all forms of dementia, VaD is considered unique because it can be prevented if the intervention is provided early enough (Thal et al., 2012). Therefore, an exploration of specific biomarkers or target genes for VaD might be of help in the development of mechanism-targeted prevention and therapy.

Although the pathologic mechanism of VaD is not yet fully elucidated, it has been reported that non-coding RNAs such as microRNAs (miRNAs), long non-coding RNAs, and circular RNAs are involved in the pathophysiology of VaD, including cognitive impairment, vascular dysfunction, neuroinflammatory response, blood-brain barrier disruption, and synaptic loss (Toyama et al., 2018; Liu et al., 2019b; Huang et al., 2020; Vijayan and Reddy, 2020; Wang et al., 2020). miRNAs are enriched in the brain and responsible for the regulation of post-transcription of a wide range of proteins through the degradation or inhibition of their target mRNAs. In a rat model of VaD, a reduction in miR-126 expression in endothelial cells revealed the pathophysiological process mediating the cognitive impairment in the rats accompanied by a decreased cerebral blood flow and vessel patency, causing the activation of astrocytes and microglia, and a reduction in synaptic plasticity (Yu et al., 2019). It has been reported that the miR-93-mediated Toll-like receptor signaling pathway plays a functional role when cognition defects in VaD is treated by acupuncture (Wang et al., 2020). In addition, the involvement of miR-124 (Liu et al., 2019a), miR-210-5p (Ren et al., 2018), miR-132 (Yang et al., 2020a), and miR-7-5p (Xu et al., 2019) has been demonstrated in the development of VaD. Although it has been proposed that miRNAs represent therapeutic targets and diagnostic biomarkers for VaD, the varying expression profiles of miRNAs and disease characteristics of VaD are not fully understood.

Almost one hundred traditional Chinese medicines are used to treat VaD, including *Ginkgo biloba* L. (Wang et al., 2013), *Salvia miltiorrhiza* Bunge (Kim et al., 2015), *Panax ginseng* C.A.Mey. (Zhu et al., 2018), and *Dracocephalum moldavica* L. (*D. moldavica*) (Jia et al., 2017; Jiang et al., 2021). Many of these herbal medicines have roles and working mechanisms in the regulation of aberrant miRNAs when used to treat vascular cognitive decline. For instance, EGb761, the standardized extract from *Ginkgo biloba* L., protects against ischemia/reperfusion-induced injury in brain microvascular endothelial cells through the lncRNA Rmst/miR-150 axis (Qiao et al., 2020). Ginsenoside Rg1 and Rg2 extracted from *Panax ginseng* C.A.Mey. have inhibitory activity on miR-155-5p (Wang et al., 2018), miR-144 (Chu et al., 2019), and miR-216a (Chen et al., 2021), thus being potential therapeutic targets in the treatment of cerebral ischemia.

*D. moldavica* is a traditional Uygur medicine in China, widely used to treat coronary heart disease, angina, and atherosclerosis as well as for calming the nerves (Dastmalchi et al., 2007; Maimaitiyiming et al., 2014; Miernisha et al., 2016). Modern neuropharmacological studies confirmed that *D. moldavica* has beneficial effects on the central nervous systems, as demonstrated by the promotion of prolonged pentobarbital-induced sedation in mice (Martinez-Vazquez et al., 2012), neuroprotection against cerebral ischemia/reperfusion injury in rats (Jia et al., 2017), and cognitive improvement against scopolamine-induced deficits in mice (Deepa et al., 2020). The total flavonoids from *D. moldavica* (TFDM) represent a standardized extract from *D. moldavica* and function as a novel treatment for VaD as established by our research team, obtaining the support of a number of major national projects in China and patent authorization. Our ongoing study demonstrated that TFDM provides neuroprotection and improvement of learning and memory capabilities against beta-amyloid-induced toxicity *in vitro* and *in vivo* (Liu et al., 2018). The principal flavonoids in TFDM, such as tilianin, luteolin, apigenin, and quercetin improve the cognitive function in various types of cognitive decline (Jiang et al., 2018 and, 2021; Liu et al., 2009; Liu et al., 2013; Zhao et al., 2013). Furthermore, TFDM and its principal compounds reduce the cognitive deficit acting on different mechanisms, including anti-oxidative, anti-amyloidogenic, neurotrophic, anti-inflammatory, and anti-apoptotic pathways as a result of the regulation of the expression of a number of genes, such as amyloid-beta precursor protein (APP), beta-secretase 1 (BACE1), cAMP-responsive element-binding protein 1 (CREB), calcium/calmodulin-dependent protein kinase II gamma (CaMKII), mitogen-activated protein kinase (MAPK), and B-cell leukemia/lymphoma-2 (Bcl-2) (Liu et al., 2009; Zhao et al., 2013; Jiang et al., 2018; Jiang et al., 2020; Liu et al., 2018). However, the effect of TFDM in the improvement of VaD through the regulation of multiple genes remains unclear. Thus, the discovery of the mechanism of action could allow its potential use in the treatment of VaD.

**FIGURE 1:**
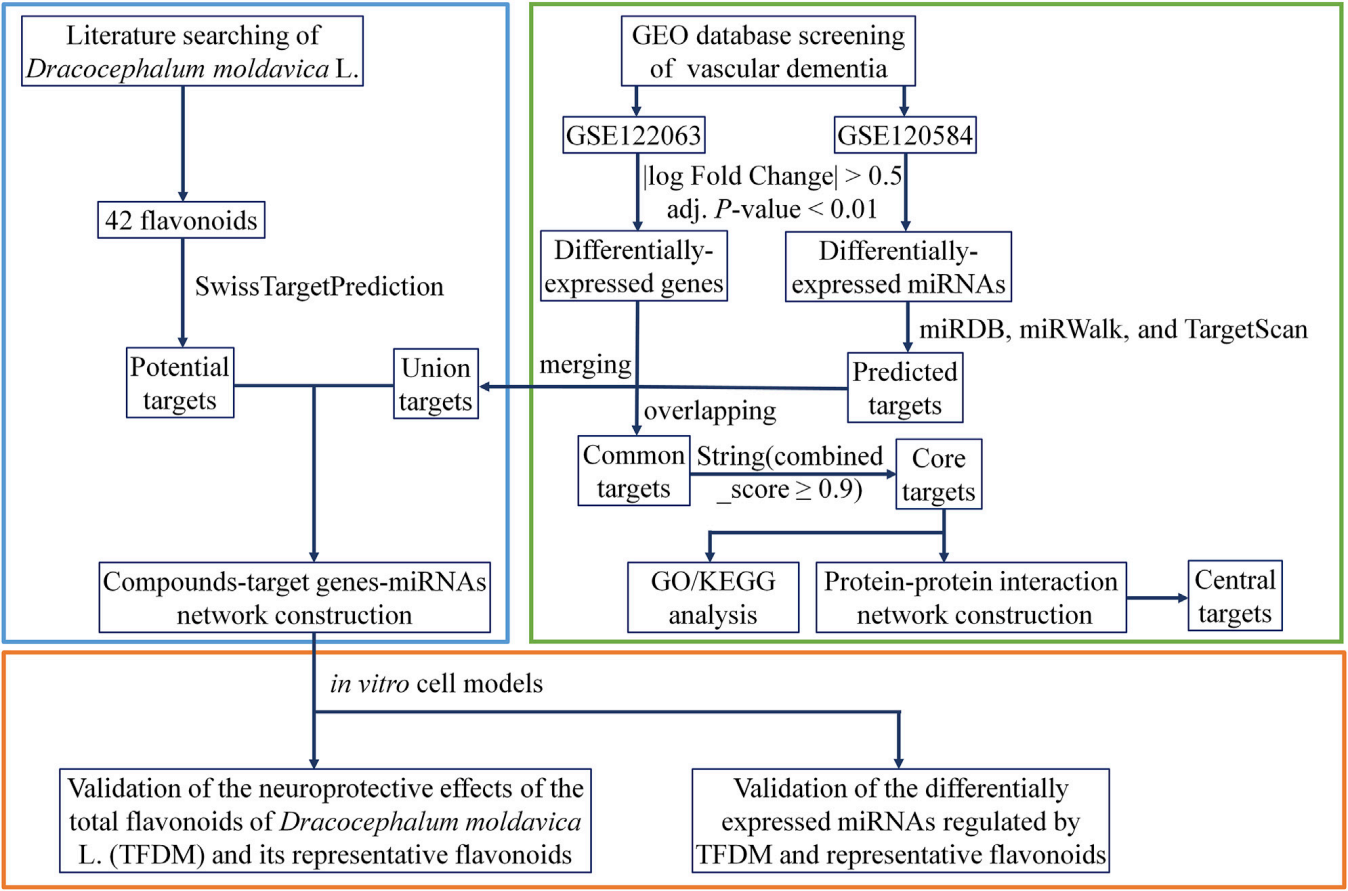
Workflow of the present study.

In the current study, bioinformatics approaches with biological verification were used to determine the miRNAs involved in the treatment of VaD by TFDM. Eight significant miRNAs that mediated 43 targets with a close interaction with TFDM were identified using the transcriptional profile network of VaD established from the Gene Expression Omnibus (GEO) database. The qRT-PCR assay revealed that the upregulation of miR-3184-3p and downregulation of miR-6875-5p might play critical roles in the treatment of VaD by TFDM. The workflow is displayed in Figure 1.

## Materials and Methods

### Dataset Acquisition

Two microarray datasets, GSE122063 (McKay et al., 2019) and GSE120584 (Shigemizu et al., 2019a), were selected from the GEO (https://www.ncbi.nlm.nih.gov/gds/) using “VaD” as the search term, with the species limited to “*Homo sapiens*”. The GSE122063 dataset includes gene expression profiles of the frontal and temporal cortex of patients with VaD, Alzheimer’s disease (AD), and non-demented controls (Control), and McKay et al. focused on the special changes in gene expression in samples of the frontal cortex of VaD patients, identifying the oxytocin receptors (OXTR) as significantly upregulated (McKay et al., 2019). In the present study, 11 samples from non-demented controls and 8 VaD samples were selected and subjected to analysis to obtain all the DEGs. Shigemizu et al. constructed risk prediction models for three subtypes of dementia, including AD, VaD, and dementia with Lewy bodies (DLB), using potential biomarkers from serum samples, and further analyzed the functional genes associated with DLB pathology (Shigemizu et al., 2019b). The samples of 91 VaD patients and normal subjects from the GSE120584 dataset were selected for miRNA analysis.

### Data Processing and Differential Expression Analysis

GEO2R (https://www.ncbi.nlm.nih.gov/geo/geo2r/) is an online analysis tool in the GEO database. It was used to screen differentially expressed genes (DEGs) between control and VaD tissue samples in the GSE122063 dataset, with a cut-off of |log Fold Change| > 0.5 and adjusted (adj.) *p*-value < 0.01. Volcano plots were generated to describe the distribution and expression of the DEGs that were identified. Differentially expressed miRNAs (DEMs) in VaD patients and normal subjects in the GSE120584 dataset were also analyzed using the GEO2R webtool. The six most upregulated and downregulated miRNAs were selected in ascending order by *p*-value.

### Target Prediction of miRNAs

The target genes of the top DEMs were predicted using miRDB (http://mirdb.org/), miRWalk (http://mirwalk.umm.uni-heidelberg.de/), and TargetScan (http://www.targetscan.org/vert_72/) online analysis tools. Overlapping target genes were identified using the Venn diagrams and then used in additional analysis. TargetScan can predict the biological target of miRNA by searching for conserved sites that match the seed region of miRNA, which are ranked by cumulative weighted context++ scores of the sites and their probability of conserved targeting. In contrast, miRDB is designed to screen target genes with a target score ≥50 by analyzing thousands of miRNA target interactions from high-throughput sequencing experiments. The miRWalk database is based on the current miRBase, using miRNA names to search for miRNA-related target gene prediction data. In the above three databases, the “human” species was selected before the prediction of the target genes.

### Gene Ontology and Kyoto Encyclopedia of Genes and Genomes Pathway Analysis

GO analysis was performed to gain insight into the molecular mechanisms of VaD by molecular function (MF), cellular component (CC), and biological process (BP) domains of the core targets derived from the overlap of target genes of the most differentially expressed miRNAs and DEGs. KEGG pathway enrichment analysis was performed to define the signaling pathways associated with these core targets. The analysis used a threshold of adj. *p*-values < 0.01.

### Construction of a Protein-Protein Interaction Network

The STRING 11.0 database (https://string-db.org/cgi/input) was used to assess the PPI network information of the core targets to discover the network of the interacting genes. A combined score of more than 0.9 was the comprehensive protein interaction score selected as the cut-off criterion. Cytoscape 3.7.0 software was then used to visualize the resulting PPI network. The four algorithms Maximal Clique Centrality (MCC), Density of Maximum Neighborhood Component (DMNC), Maximum Neighborhood Component (MNC), and DEGREE in the cytohubba plug-in were used to identify standard and central targets with high connectivity in the gene expression network.

### Construction of Compound-Target Gene-miRNA Network for TFDM

#### Identification of Components and Target Screening

The flavonoids in *D. moldavica* were searched in the reported literature (Yang et al., 2013; Liu et al., 2019c; Wu et al., 2020). Then, query Canonical SMILES of flavonoids in Pubchem was used to predict potential target genes. The potential targets of these compounds were predicted with probability ≥0.05 using the online prediction tool SwissTargetPrediction (http://www.swisstargetprediction.ch/). This portal allows the estimation of the most likely macromolecular targets for small molecules considered biologically active. The prediction was based on the similarity of the 2D and 3D compounds.

### Network Construction

Compound-target (C-T) and compound-target gene-miRNA (C-T-M) networks were constructed using Cytoscape. The eigenvector centrality parameter of the node topology was calculated using Gephi 0.9.2 software by the analysis of the importance of each node, which depends on the number of nodes of its neighbours and their importance. Therefore, it is considered an appropriate predictor in interactive networks. In the network, the nodes represent the compounds, the targets, and the miRNAs, while the edges represent the relationship between compounds and targets or between targets and miRNAs.

### Drug Materials

#### Plant Source

*D. moldavica* from the Lamiaceae Martinov family belongs to the genus of *Dracocephalum L*. The samples used in the present work were produced in Jimusaer (Xinjiang province, China) in July 2017 (batch number: 20170713). The *D. moldavica* specimen (D170713) was deposited in the herbarium of the Xinjiang Institute of Materia Medica (Ürümqi, China) and identified by Prof. Jiang He, Xinjiang Institute of Materia Medica (Ürümqi, China).

#### Extraction of TFDM and Isolation of Tilianin

After confirmation of the herb, the powder of the aboveground parts (90 kg) was airdried at room temperature (RT), then refluxed three times with 40% EtOH at 100°C. Next, 3.2 kg crude extract was collected after evaporation of the filtered ethanol solution under reduced pressure. The crude extract was partitioned by column chromatography with a HPD600 resin and eluted with water, 50% EtOH, and 70% EtOH. The eluent was concentrated and dried to obtain TFDM, which was evaluated by high-performance liquid chromatography (HPLC).

The HPLC analysis was performed on a Sthermos U3000 HPLC system (Thermo Fisher Scientific, PA, USA). All separations were performed on a Welch Ultimater XB-C18 column (250 × 4.6 mm, 5 μm), with an injection volume of 20 μL. The mobile phase was composed of acetonitrile A and 0.5% formic acid aqueous solution B using a specific gradient elution (0–60 min, 20% A; 60–69 min, 50% A; 69–70 min, 80% A; 70–80 min, 20% A). The flow rate of the mobile phase was 1.0 ml min^−1^, and the temperature was maintained at 35°C. The components were quantified by the peak areas at 330 nm UV wavelength.

The purified product was collected by filtering the 70% EtOH eluate on a silica gel column (100–200 mesh, chloroform:methanol, 95:5–90:10–80:20) to remove the impurities. The structure of the compound was subsequently determined by the physicochemical and spectroscopic data, and the purity of the compound was 99% as revealed by HPLC analysis, yielding a total of 280 mg of tilianin, which was the same batch in our previous research (Liu et al., 2018).

#### Preparation of the Pharmaceutical Solution

Apigenin (4,5,7-trihydroxyflavone, 97%) and luteolin (3′,4′,5,7-tetrahydroxyflavone, 98%) used in this work were purchased from Acmec Biochemical (Shanghai, China). TFDM and other three active compounds were dissolved in dimethyl sulfoxide (DMSO, Sigma-Adrich, St. Louis, MO, USA) to a stock concentration of 100 mM and kept at a temperature of −20°C in the dark. The final dilution was freshly prepared at the time of the treatment by diluting the concentrated solution directly into the culture medium.

### Cell Culture and Treatment

SH-SY5Y cells (ATCC; Manassas, VA, USA) were routinely cultured in Dulbecco’s Modified Eagle Medium (DMEM; Gibco, Grand Island, NY, USA) supplemented with 10% fetal bovine serum (FBS; Gibco, Grand Island, NY, USA). Cells were seeded in 96-well plates at a density of 1 × 10^4^ cells/well in sugar-free DMEM and subjected to the hypoxic condition of 93.7%N_2_/1.3%O_2_/5%CO_2_ for 24 h at 37°C in a tri-gas incubator (Wiggens, Straubenhardt, Germany) for the establishment of an *in vitro* OGD model. Cells were treated with different concentrations of TFDM (25–100 μg/ml), tilianin (8–32 μM), luteolin (2.5–10 μM), and apigenin (2.5–10 μM) in the standard medium in a normoxic condition for 12 h prior to OGD injury. Next, the medium was replaced with the same sugar-free one with the same compounds at the same concentration during the OGD process.

### Cell Viability Assay

Cell survival was assessed using the cell counting kit-8 (CCK8; Vazyme Biotech, Nanjing, China) according to the manufacturer’s instructions and measured at 450 nm using a Spark 20M multimode microplate reader (Tecan Group Ltd., Mannedorf, Switzerland).

### Expression of DEMs by Quantitative Reverse Transcription-Polymerase Chain Reaction

The qRT-PCR was used to confirm the changes of the predicted DEMs in the OGD-injured SH-SY5Y cells treated with TFDM and principal compounds. Briefly, total RNA was extracted using Trizol reagent (Invitrogen). miRNA was reverse-transcribed using a miRNA 1st Strand cDNA Synthesis Kit (by stem-loop) (Vazyme Biotech). PCR was performed using miRNA Universal SYBR qPCR Master Mix (Vazyme Biotech). Relative miRNA expression was calculated using the 2^−ΔΔCT^ method. Results were normalized using small nuclear U6 RNA as the internal control. Primers are listed in Table 1.

**TABLE 1:** List of primers used for qRT-PCR.

miRNA	Sequences
miR-3184-3p	RT prime: GTC​GTA​TCC​AGT​GCA​GGG​TCC​GAG​GTA​TTC​GCA​CTG​GAT​ACG​ACT​GAG​GG
	Forward: GCG​AAA​GTC​TCG​CTC​TCT​GC
	Reverse: AGT​GCA​GGG​TCC​GAG​GTA​TT
mir-6875-5p	RT prime: GTC​GTA​TCC​AGT​GCA​GGG​TCC​GAG​GTA​TTC​GCA​CTG​GAT​ACG​ACT​CTC​CT
	Forward: GCGTGAGGGACCCAGGAC
	Reverse: AGT​GCA​GGG​TCC​GAG​GTA​TT
miR-6762-3p	RT prime: GTC​GTA​TCC​AGT​GCA​GGG​TCC​GAG​GTA​TTC​GCA​CTG​GAT​ACG​ACC​TGG​AG
	Forward: CGT​GGC​TGC​TTC​CCT​TGG​T
	Reverse: AGT​GCA​GGG​TCC​GAG​GTA​TT
miR-6777-3p	RT prime: GTC​GTA​TCC​AGT​GCA​GGG​TCC​GAG​GTA​TTC​GCA​CTG​GAT​ACG​ACC​TGG​GG
	Forward: CGCGTCCACTCTCCTGGC
	Reverse: AGT​GCA​GGG​TCC​GAG​GTA​TT
miR-6784-3p	RT prime: GTC​GTA​TCC​AGT​GCA​GGG​TCC​GAG​GTA​TTC​GCA​CTG​GAT​ACG​ACC​TGG​GG
	Forward: GCG​TCT​CAC​CCC​AAC​TCT​G
	Reverse: AGT​GCA​GGG​TCC​GAG​GTA​TT

### Statistical Analysis

Statistical analysis was performed using GraphPad Prism Version 7.0 software (GraphPad Prism Software; La Jolla, CA, USA). Data were analyzed using one-way ANOVA, followed by a Tukey’s multiple comparison test, or Student’s *t*-test. The results were expressed as mean ± standard deviation (SD). *p*-values less than 0.05 were considered statistically significant.

## Results

### Identification of DEGs in VaD

**FIGURE 2:**
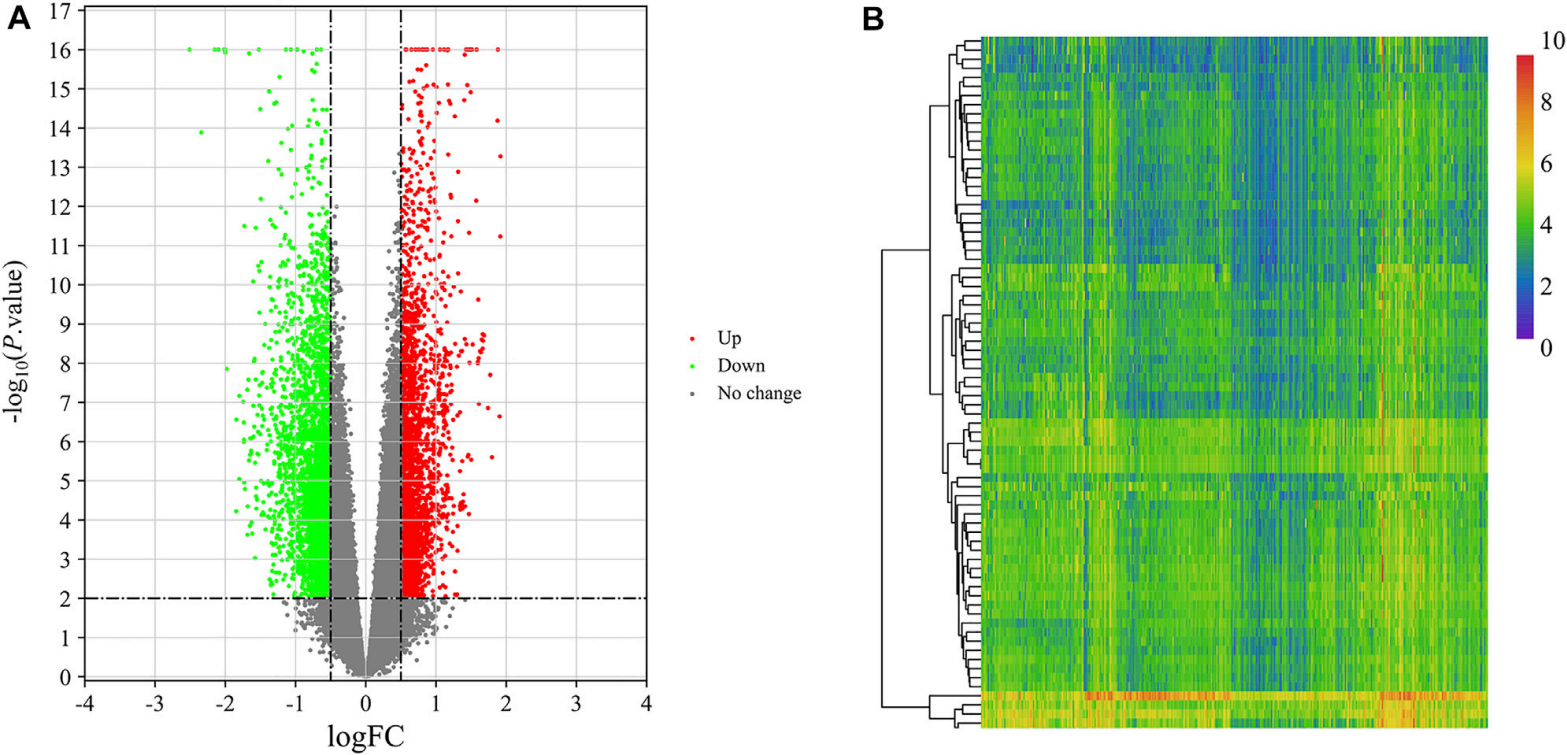
Identification of DEGs and DEMs in VaD. **(A)** Volcano plot of GSE122063 for the analysis of DEGs. **(B)** Heat map of the identified DEMs from the GSE120584 dataset.

DEGs in VaD were analyzed in the GSE122063 dataset. Using the cut-off criteria |log Fold Change| > 0.5 and adj. *p*-value < 0.01, 1,478 significantly upregulated DEGs were identified and 2,044 were downregulated in the brains of patients with VaD compared with normal control brains, as presented in Figure 2A. The 15 most downregulated and upregulated genes whose expression changed significantly are listed in Table 2. The genes with a significantly increased expression included Nexin 31 (SNX31), Fc Fragment of IgG Binding Protein (FCGBP), SLAM Family Member 8 (SLAMF8), Maternal immune activation (MIA), and XLOC_001219, and those with a significantly decreased expression were RNA Binding Motif Protein 3 (RBM3), XLOC_006951, stathmin 1 (STMN1), LOC100506274, and chaperonin Containing TCP1 Subunit 6B (CCT6B).

**TABLE 2:** Fifteen most upregulated and downregulated DEGs.

Downregulated gene (SYMBOL)	*P*. Value	adj.*P*. Val	logFC	Upregulated gene (SYMBOL)	*P*. Value	adj.*P*. Val	logFC
RBM3	1.00E-22	1.18E-18	−2.510	SNX31	5.28E-14	2.54E-11	1.918
XLOC_006951	1.30E-14	7.58E-12	−2.342	FCGBP	5.85E-12	1.29E-09	1.915
STMN1	7.48E-19	1.76E-15	−2.150	SLAMF8	2.28E-07	6.60E-06	1.906
LOC100506274	1.80E-19	5.28E-16	−2.097	MIA	6.26E-19	1.53E-15	1.881
CCT6B	2.46E-19	6.28E-16	−2.015	XLOC_001219	6.50E-15	4.24E-12	1.876
PBOV1	1.40E-08	6.86E-07	−1.980	CD163	2.50E-06	4.67E-05	1.797
LINC00458	2.75E-07	7.75E-06	−1.842	VSIG4	1.98E-08	9.18E-07	1.772
XLOC_006657	9.01E-06	1.34E-04	−1.801	SIGLEC14	1.39E-07	4.42E-06	1.740
XLOC_011468	6.82E-08	2.46E-06	−1.800	SPP1	1.95E-09	1.42E-07	1.684
PI3	9.66E-08	3.28E-06	−1.744	RNASE2	1.08E-07	3.60E-06	1.610
LOC100505994	1.02E-06	2.24E-05	−1.736	AQP1	2.38E-10	2.53E-08	1.602
CRH	3.27E-07	8.89E-06	−1.736	HSPA1A	2.95E-18	5.98E-15	1.579
XLOC_001329	8.93E-07	2.02E-05	−1.734	C1QC	7.16E-13	2.26E-10	1.572
XLOC_001406	1.11E-05	1.59E-04	−1.731	C1QB	3.32E-09	2.19E-07	1.522
XLOC_013968	3.16E-12	7.94E-10	−1.729	MYBPH	2.90E-06	5.31E-05	1.501

### Identification of DEMs in VaD

The miRNA dataset GSE120584 was screened for DEMs in VaD using the GEO2R web tool to compare VaD patients with the normal group. A total of 2,562 DEMs were identified from this microarray dataset, including 1,167 upregulated miRNAs and 1,395 downregulated miRNAs, as shown in a heat map (Figure 2B). The six most upregulated miRNAs were hsa-miR-3184-5p, hsa-miR-4667-5p, hsa-miR-6875-5p, hsa-miR-4746-3p, hsa-miR-4467, and hsa-miR-1268a, while the six most downregulated miRNAs were hsa-miR-6777-3p, hsa-miR-6784-3p, hsa-miR-3184-3p, hsa-miR-6762-3p, hsa-miR-4747-3p, and hsa-miR-3675-3p, as listed in Table 3.

**TABLE 3:** Six most upregulated and downregulated DEMs.

Downregulated miRNA (ID)	adj.P. Val	*P*. Value	logFC	Upregulated miRNA (ID)	adj.*P*. Val	*P*. Value	logFC
hsa-miR-6777-3p	8.23E-14	3.21E-17	−0.347366	hsa-miR-3184-5p	1.65E-06	1.12E-07	0.322646
hsa-miR-6784-3p	1.88E-11	1.47E-14	−0.422261	hsa-miR-4667-5p	7.16E-06	6.34E-07	0.234238
hsa-miR-3184-3p	3.01E-11	3.80E-14	−0.579104	hsa-miR-6875-5p	2.43E-05	2.48E-06	0.261412
hsa-miR-6762-3p	3.01E-11	4.71E-14	−0.67333	hsa-miR-4746-3p	8.04E-05	9.72E-06	0.2373
hsa-miR-4747-3p	4.07E-11	7.95E-14	−0.677231	hsa-miR-4467	1.68E-04	2.22E-05	0.253021
hsa-miR-3675-3p	5.17E-11	1.21E-13	−0.444784	hsa-miR-1268a	1.75E-04	2.32E-05	0.145002

### Target Prediction, Core Target Analysis, and Functional Analysis

**FIGURE 3:**
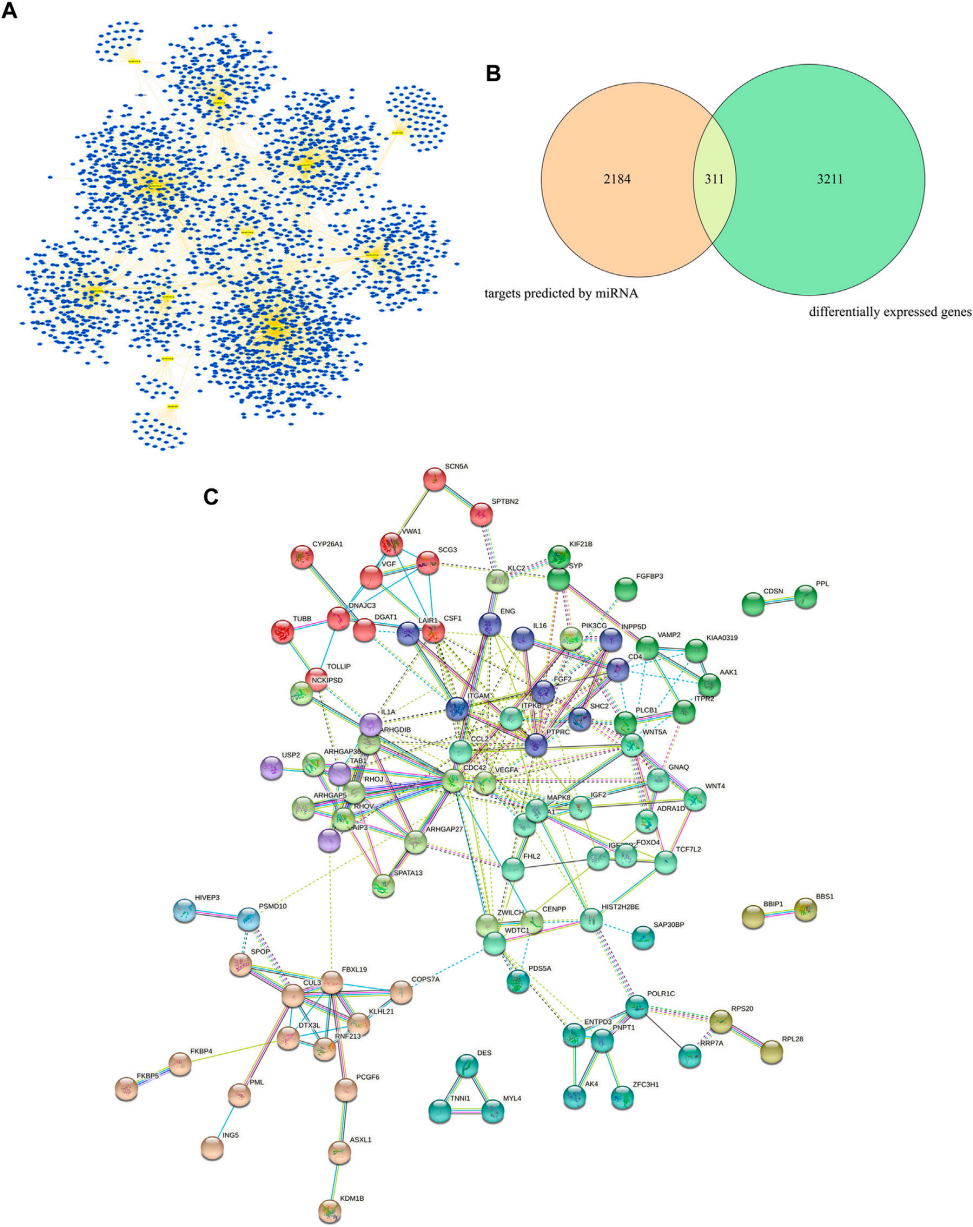
Target prediction, core target analysis, and functional analysis of DEGs and DEMs in VaD. **(A)** miRNA-gene regulatory network of the 12 most dysregulated DEMs and their predicted target genes. **(B)** Venn diagram of the intersection between miRNA-predicted targets and DEGs. **(C)** Protein-protein interaction analysis of 94 core targets using Cytoscape.

The target genes of the 12 most dysregulated DEMs were predicted using the online analysis tools miRDB, miRWalk, and TargetScan. A total of 2,495 targets were identified. The miRNA-gene regulatory network was mapped (Figure 3A) using Cytoscape software. Next, the screening was performed using the parameters |log Fold Change| > 0.5 and adj. *p*-value < 0.01, and 3,522 DEGs were identified. A total of 311 common targets were identified by analyzing the association between DEGs and the target genes of miRNAs (Figure 3B), considered core targets closely related to the principal pathophysiological process of VaD. STRING was then used to analyze the core targets, resulting in a total of 94 core targets with a combined_score ≥0.9, which were selected for further analysis. They were imported into Cytoscape 3.7.0 and the key associations were studied. The complex PPI network contained 94 nodes and 228 edges (Figure 3C).

**FIGURE 4:**
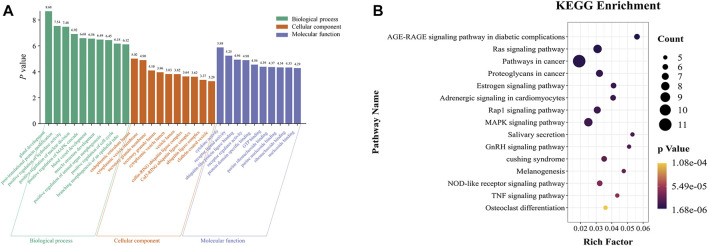
GO and KEGG pathway analysis of the 94 core targets. **(A)** Top 10 significantly enriched GO terms. **(B)** Top 15 significant KEGG pathway terms.

GO and KEGG pathway analyses were used to determine the enrichment of biological function and the pathways of these 94 core target genes. The GO annotation revealed that most significantly enriched pathways in BP were mostly enriched in post-translational protein modification, positive regulation of hydrolase activity, positive regulation of the MAPK cascade, and blood vessel development. The most significantly enriched pathways in MF were cytokine activity, receptor-ligand activity, ubiquitin-like protein ligase binding, and purine ribonucleoside binding. The CC terms were mainly represented by cytoplasmic vesicle membrane, secretory granule membrane, and cullin-RING ubiquitin ligase complex (Figure 4A).

The signaling pathways enriched in KEGG analysis were ranked in ascending order according to the *p*-value. The results revealed that the selected core targets were significantly enriched in the advanced glycation end products (AGE) - receptor for AGE (RAGE) signaling pathway in diabetic complications, Ras signaling pathway, estrogen signaling pathway, Rap1 signaling pathway, MAPK signaling pathway, tumor necrosis factor (TNF) signaling pathway, and other signaling pathways (Figure 4B).

### Construction of a PPI Network of Central DEGs

**FIGURE 5:**
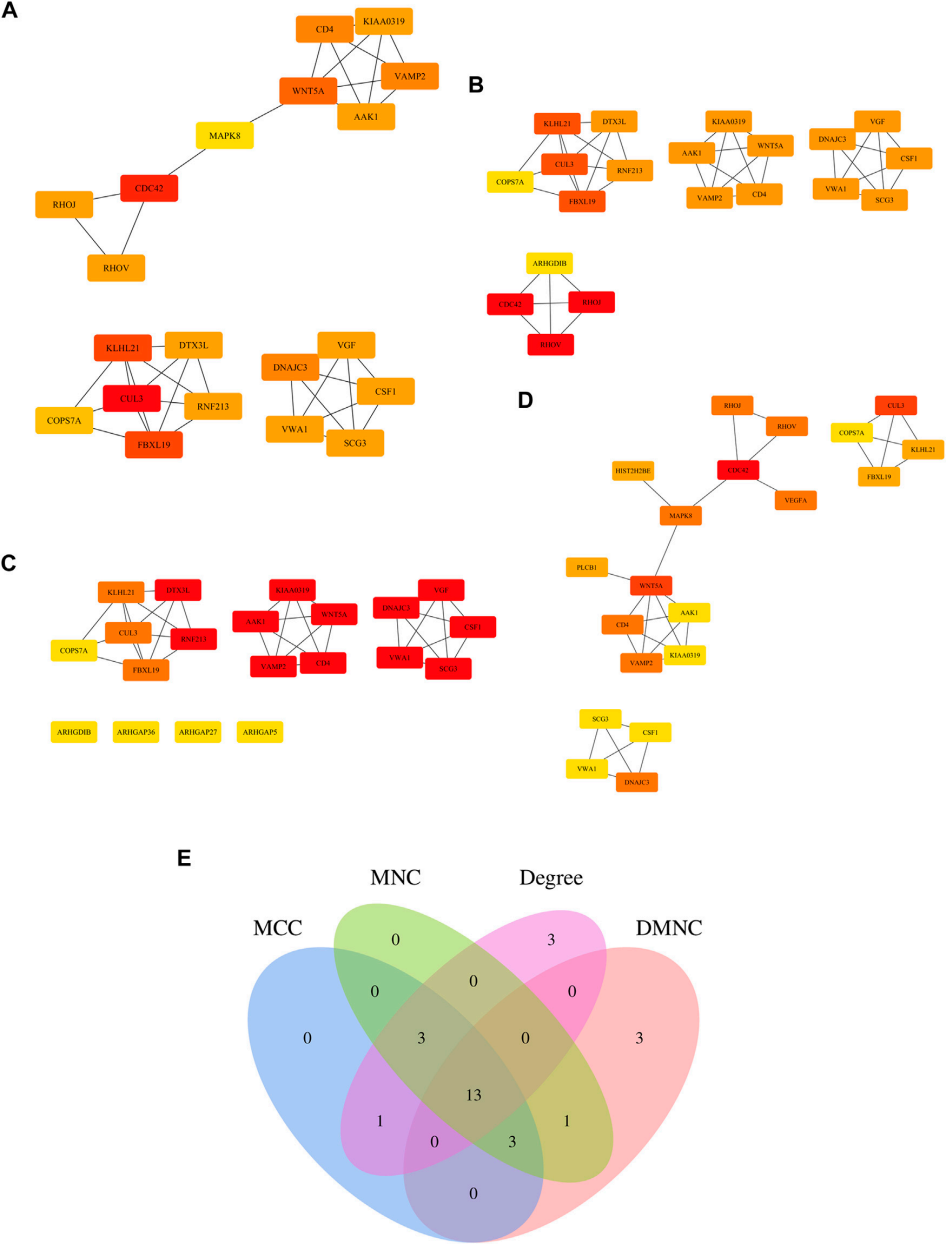
Determination of the central genes of the 94 core targets. **(A–D)** Four different metrics were used, such as Maximal Clique Centrality (MCC) **(A)**, Density of Maximum Neighborhood Component (DMNC) **(B)**, Maximum Neighborhood Component (MNC) **(C)**, and DEGREE **(D)** to analyze central genes of the 94 core targets. **(E)** Venn diagram showing 13 overlapping central genes from these four metrics.

A number of central targets based on the MCC (Figure 5A), DMNC (Figure 5B), MNC (Figure 5C), and DEGREE (Figure 5D) algorithms in Cytoscape software were identified from the extraction of 94 core target gene-related proteins in VaD. Thirteen central targets with a high degree of connectivity were finally identified, such as Cullin 3 (CUL3), kelch like family member 21 (KLHL21), F-box and leucine-rich repeat protein 19 (FBXL19), Wnt family member 5A (WNT5A), DnaJ homolog subfamily C member 3 (DNAJC3), cluster of differentiation 4 (CD4), vesicle-associated membrane protein 2 (VAMP2), AP2-associated protein kinase 1 (AAK1), Dyslexia-associated protein KIAA0319 (KIAA0319), colony-stimulating factor 1 (CSF1), Von Willebrand factor A domain containing 1 (VWA1), Secretogranin III (SCG3), and COP9 Signalosome Subunit 7A (COPS7A) (Figure 5E).

### Construction of a C-T-M Network for TFDM

**FIGURE 6:**
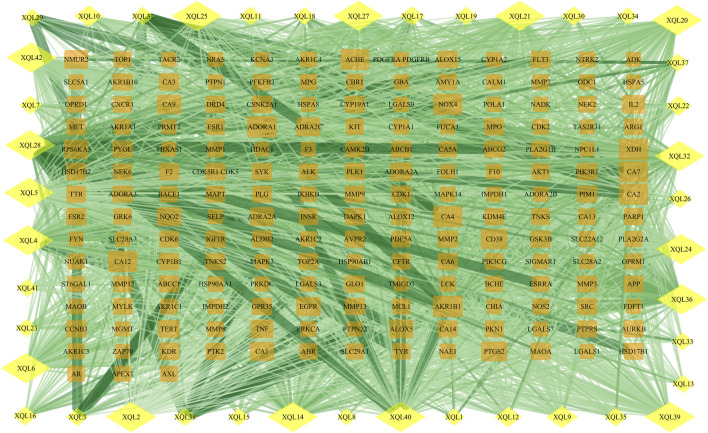
Protein network of TFDM-related targets. Yellow diamonds represent flavonoids, and orange rectangles represent potential targets of the compounds.

**FIGURE 7:**
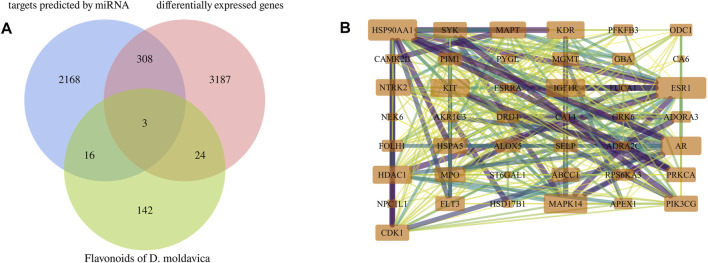
Identification of key targets for TFDM. **(A)** Intersection Venn diagram of potential targets of VaD and targets of TFDM. **(B)** Forty-three effective targets of TFDM for the treatment of VaD overlapped in the established potential VaD target library.

**FIGURE 8:**
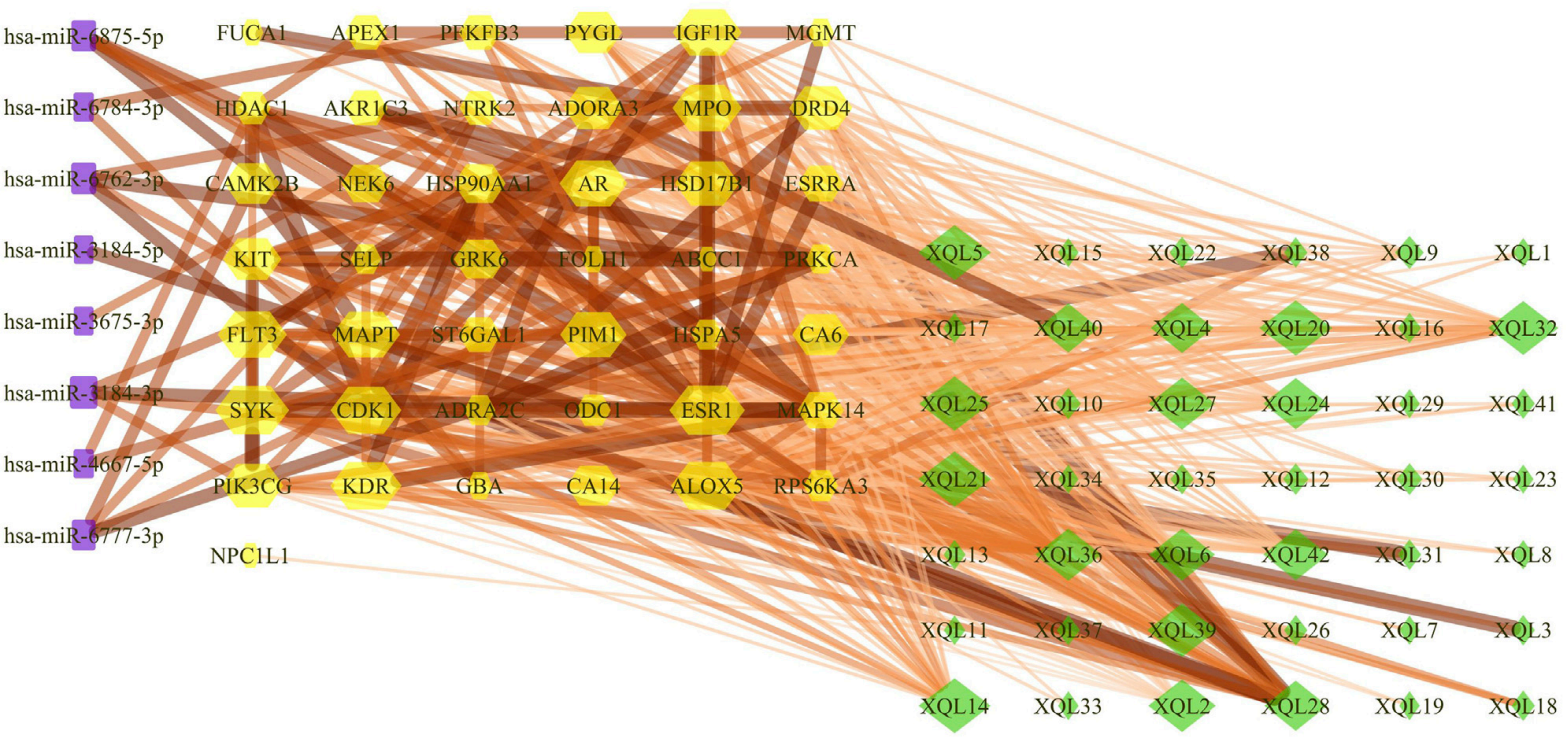
Compound-target gene-miRNA (C-T-M) network. The purple rectangle represents the miRNAs, the yellow hexagon represents the targets, and the green diamond represents the flavonoids of TFDM.

*D. moldavica* contains 42 flavonoids, including kaempferol, chrysoeriol, diosmetin, 8-hydroxy-salvigenin, apigenin, luteolin, salvigenin, 3-hydroxyflavone, isorhamnetin, quercetin, acacetin, gardenin A/B, and thymonin, as shown in Table 4. A total of 185 targets for TFDM were screened by target prediction using SwissTargetPrediction (Figure 6). Prior to the prediction of potential VaD-related targets for TFDM, a combination of 2,495 targets predicted by the 12 most dysregulated miRNAs and 3,522 differentially expressed target genes resulted in 5,706 combined targets that were considered potential targets and comprised a library related to the pathophysiology of VaD. Subsequently, genes common to both the potential VaD target library and targets predicted for TFDM were analyzed and selected (Figure 7A), yielding 43 common targets, classified as key targets for TFDM (Figures 7A,B), and considered those effective for the treatment of VaD. Furthermore, the corresponding miRNAs that bind to the common targets were identified and analyzed, and the results indicated that 8 miRNAs mediated 43 targets that potentially contributed to the neuroprotective effects of TFDM (consisting of 42 flavonoid compounds) on VaD (Figure 8). The 15 most active compounds, 15 vital mRNAs, and 8 miRNAs were found from the list of eigenvector centrality in descending order, as listed in Table 5.

**TABLE 4:** Information including name, compound CID, number of predicted targets, and compound ID used in the present study on the 42 flavonoids in *Dracocephalum moldavica* L.

Name	Compound CID	Number of predicted targets	Compound ID
Chrysoeriol	5280666	100	XQL20
Diosmetin	5281612	100	XQL21
8-Hydroxy-Salvigenin	3083783	100	XQL5
Apigenin	5280443	100	XQL14
Gardenin B	96539	100	XQL25
Luteolin	5280445	100	XQL32
Salvigenin	161271	100	XQL39
3-Hydroxyflavone	11349	100	XQL2
Isorhamnetin	5281654	100	XQL27
Quercetin	5280343	100	XQL36
Acacetin	5280442	100	XQL6
Gardenin A	261859	100	XQL24
Thymonin	442662	100	XQL42
Kaempferol	5280863	100	XQL28
5,7,4′-trihydroxy-3′methoxy flavone		100	XQL4
Scrophulein	188323	100	XQL40
Acacetin 7-(6″-acetylglucoside)	52929806	47	XQL9
Acacetin 7-Glucoside	44257884	35	XQL12
Acacetin 7-Glucuronide	44257886	21	XQL13
Acacetin-6-Glucuronide		13	XQL8
Tilianin\Acacetin 7-O-Glucoside	5321954	23	XQL7
Buddleoside\Acacetin-7-O-Rutinoside	5317025	23	XQL19
Acacetin-7-O-β-D-(6-O-malonyl) Glucoside		24	XQL11
Acacetin-7-O-β-D-(4″-acetyl)-glucopyranoside		42	XQL10
Kaempferol-3-O-β-D-(6″-O-p-coumaroyl)-glucopyranoside	12775850	30	XQL30
Kaempferol-7-O-β-D-(6″-O-p-coumaroyl)-gIucopyranoside		22	XQL31
Kaempferol-3-O-β-D-(6″-O-pcoumaroyl)-galactoside		18	XQL29
Apigenin 7-Galactoside	44257799	27	XQL16
Apigenin 7-glucoside	5280704	31	XQL17
Apigenin-7-O-β-D-(6″-Malonyl)-Glucopyranoside		18	XQL15
Diosmetin 7-O-beta-D-glucopyranoside	11016019	31	XQL22
DiosMetin 7-O-beta-D-Glucuronide	54462250	22	XQL23
5,7,3,'4′-tetrahydroxy-3-α-β-Glu-Rha flavone		21	XQL3
Astragalin	5282102	20	XQL18
Homoplantaginin	5318083	25	XQL26
Luteolin 7-Glucoside	5280637	25	XQL35
Luteolin 7-Glucuronide	5280601	19	XQL33
Luteolin 7-O-(6-O-Malonyl- -D-Glucoside)	419581190	17	XQL34
Quercetin-3-O-[α-Lrhamnopyranosyl (1→6)]-β-D-Glucopyranoside		21	XQL37
Quercetin 3-glucoside	5280804	22	XQL38
Takakin 8-O-beta-D-glucoside	44258581	22	XQL41
2″-P-Coumarylastragalin	6442291	17	XQL1

**TABLE 5:** Correspondence of top 15 compounds, target genes, and miRNAs based on eigenvector centrality within the predicted compound-mRNA-miRNA network of total flavonoids from *Dracocephalum moldavica* L. (TFDM).

miRNA ID	Eigenvector centrality	Compound ID	Compound name	Eigenvector centrality	Gene symbol	Gene name	Eigenvector centrality
miR-3184-3p	0.156643	XQL20	Chrysoeriol	0.957493	ESR1	Estrogen receptor alpha	1
miR-6762-3p	0.114508	XQL21	Diosmetin	0.957493	SYK	Spleen tyrosine kinase	0.968225
miR-6875-5p	0.105428	XQL5	8-hydroxy-salvigenin	0.950177	ALOX5	Arachidonate 5-lipoxygenase	0.942781
miR-6777-3p	0.09075	XQL14	Apigenin	0.941078	KDR	Vascular endothelial growth factor receptor 2	0.926816
miR-6784-3p	0.030171	XQL25	Gardenin B	0.922549	CDK1	Cyclin-dependent kinase 1	0.918295
miR-4667-5p	0.029771	XQL32	Luteolin	0.905914	MPO	Myeloperoxidase	0.892825
miR-3675-3p	0.02028	XQL39	Salvigenin	0.90154	FLT3	FMS-like tyrosine kinase 3	0.89067
miR-3184-5p	0.015082	XQL2	3-Hydroxyflavone	0.860698	IGF1R	Insulin-like growth factor I receptor	0.888558
		XQL27	Isorhamnetin	0.840028	MAPT	Microtubule-associated protein tau	0.868808
		XQL36	Quercetin	0.840028	AR	Androgen Receptor	0.865538
		XQL6	Acacetin	0.833531	PIM1	Serine/threonine-protein kinase PIM1	0.841692
		XQL24	Gardenin A	0.810614	HSD17B1	Estradiol 17-beta-dehydrogenase 1	0.804947
		XQL42	Thymonin	0.810614	DRD4	Dopamine D4 receptor	0.783832
		XQL28	Kaempferol	0.777624	PIK3CG	PI3-kinase p110-gamma subunit	0.77247
		XQL4	5,7,4′-trihydroxy-3′methoxy flavone	0.776246	ADORA3	Adenosine A3 receptor	0.719892

### Neuroprotection and Verification of DEMs Regulated by TFDM

**FIGURE 9:**
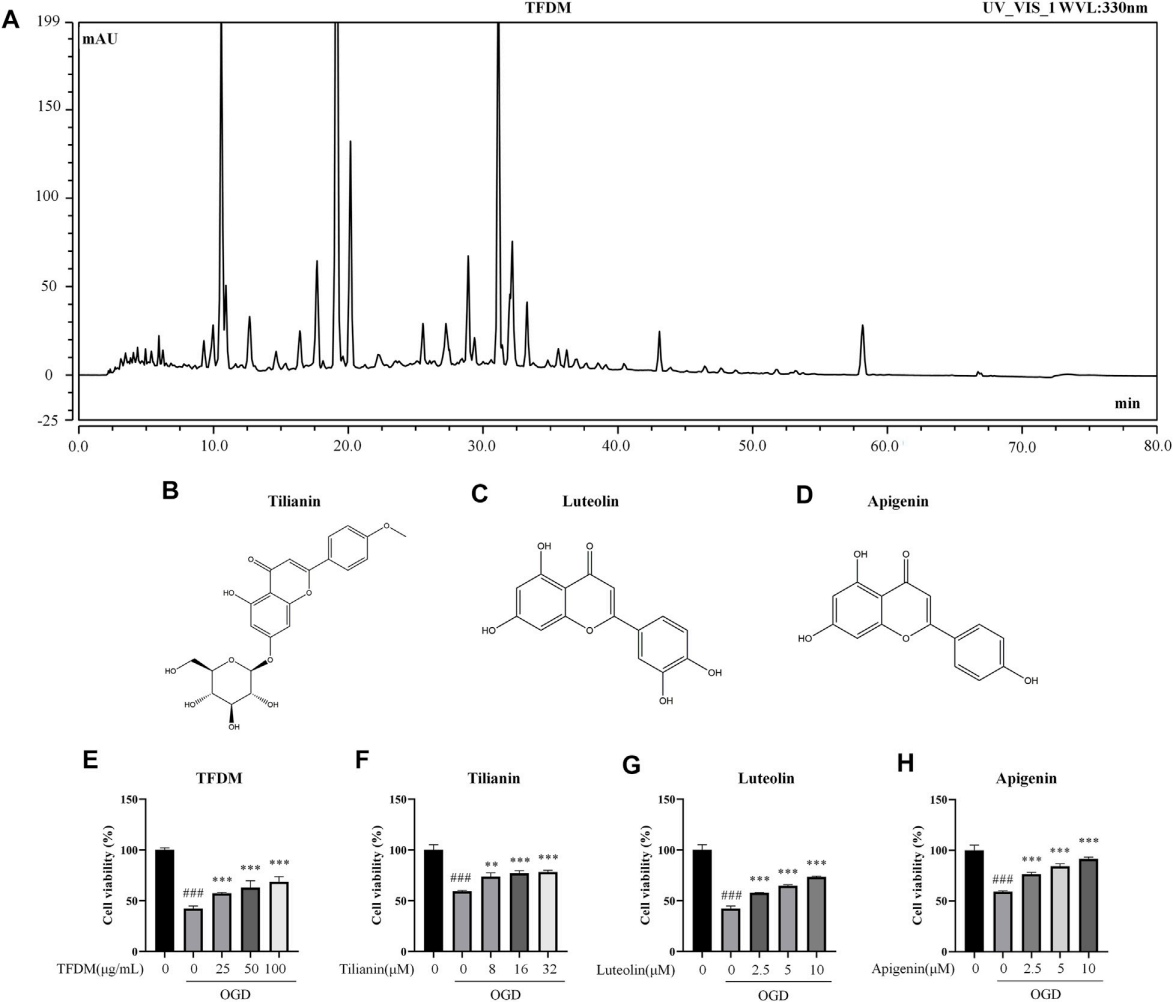
Fingerprint of TFDM, compound structures, and neuroprotective effects of TFDM as well as active compounds on neuronal cells against OGD injury. **(A)** HPLC chromatogram at 330 nm for TFDM. **(B–D)** Structure of tilianin **(B)**, luteolin **(C)**, and apigenin **(D)**. **(E)** Improved cell viability of SH-SY5Y cells by TFDM after the exposure to OGD. **(F–H)** Improved cell viability of SH-SY5Y cells by tilianin **(F)**, luteolin **(G)**, and apigenin **(H)** after the exposure to OGD. Statistical analysis was performed using one-way ANOVA followed by a Tukey’s multiple comparison test. *n* = 3. ^###^ *p* < 0.001 vs*.* control; **p < 0.01, ***p < 0.001 vs*.* OGD. Results are expressed as mean ± SD.

Considering our previous research on TFDM, this compound and its representative flavonoids, including tilianin, luteolin, and apigenin, were selected to investigate the effects on the expression of predicted miRNAs. The fingerprint (approximately 71 peaks) of TFDM and the structure of tilianin, luteolin, and apigenin are shown in Figures 9A–D.

Before the verification of the predicted miRNAs regulated by TFDM, the neuroprotective effects of TFDM and its representative flavonoids on OGD-injured SH-SY5Y cells were measured. TFDM significantly improved the cell viability of OGD-injured SH-SY5Y cells in a dose-dependent manner at 25 μg/ml, 50 μg/ml, and 100 μg/ml (all *p* < 0.001), as shown in Figure 9E. The three active compounds from TFDM, such as tilianin, luteolin, and apigenin, also increased cell viability at the selected concentrations (*p* < 0.01–0.001, Figures 9F–H). These results suggested that TFDM and its three active compounds had neuroprotective effects against OGD-induced injury.

The relationship of the C-T-M network revealed that miR-3184-3p was a potential miRNA targeted by each flavonoid among the eight miRNAs. In addition, miR-6875-5p, miR-6777-3p, miR-6762-3p, and miR-6784-3p were targeted by 37, 17, 16, and 12 flavonoids from TFDM, respectively, suggesting that these five miRNAs might play a role in the treatment of VaD by TFDM. Therefore, miR-3184-3p, miR-6875-5p, miR-6777-3p, miR-6762-3p, and miR-6784-3p were selected to be confirmed in OGD-injured SH-SY5Y cells treated by TFDM.

**FIGURE 10:**
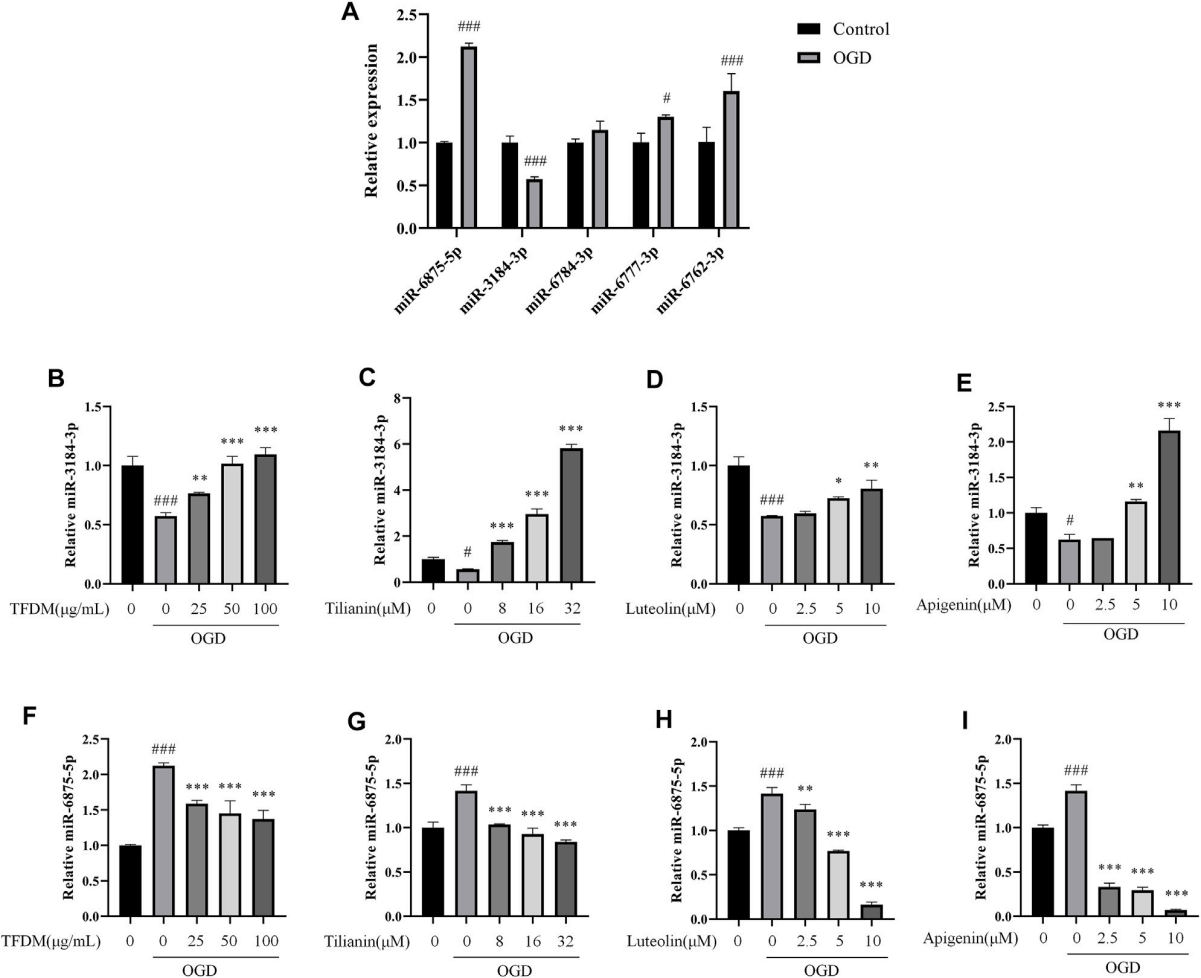
Regulation of miR-3184-3p and miR-6875-5p by TFDM and active compounds in OGD-injured SH-SY5Y cells. **(A)** Changes in the expression of miRNAs in OGD-injured SH-SY5Y cells compared with control cells. **(B–E)** Upregulation of miR-3184-3p in OGD-injured SH-SY5Y cells after the treatment with TFDM **(B)** and the active compounds tilianin **(C)**, luteolin **(D)**, and apigenin **(E)**. **(F–I)** Downregulation of miR-6875-5p in OGD-injured SH-SY5Y cells after the treatment with TFDM **(F)** and the active compounds tilianin **(G)**, luteolin **(H)**, and apigenin **(I)**. Statistical analysis was performed using Student’s *t*-test or one-way ANOVA followed by a Tukey’s multiple comparison test. *n* = 3. ^###^ *p* < 0.001 vs*.* control; *p < 0.05, **p < 0.01, ***p < 0.001 vs*.* OGD. Results were expressed as mean ± SD.

According to the qRT-PCR results, the expression of miR-3184-3p was decreased, and miR-6875-5p was increased in response to the OGD injury (both *p* < 0.001, Figure 10A), consistent with the analysis of the C-T-M network. The exposure to OGD did not significantly changed the expression of miR-6784-3p compared with the control group (Figure 10A), while the expression of miR-6777-3p and miR-6762-3p was increased compared with the control group (*p* < 0.05 and 0.001), which was an opposite result compared to the C-T-M network. Thus, these results indicated that miR-3184-3p and miR-6875-5p were confirmed as having a significant aberrant change in the process of cerebral ischemia, which was consistent with previous bioinformatics analysis.

Furthermore, miR-3184-3p was upregulated and miR-6875-5p was downregulated by TFDM treatment in a dose-dependent manner at 25 μg/ml, 50 μg/ml, and 100 μg/ml in OGD-injured SH-SY5Y cells compared with control cells (*p* < 0.01–0.001, Figures 10B,F), accompanied by the increase of cell viability. In the same way, tilianin, luteolin, and apigenin increased the expression of miR-3184-3p and decreased the expression of miR-6875-5p in OGD-injured SH-SY5Y cells compared with control cells (*p* < 0.05–0.001, Figures 10C–E and G-I). Therefore, these results suggested that miR-3184-3p and miR-6875-5p might play a role in the treatment of VaD by TFDM.

## Discussion

To our knowledge, the present study is the first constructing a C-T-M network indicating the potential molecular mechanism of TFDM on the treatment of VaD. Subsequent confirmation provided evidence that miR-3184-3p and miR-6875-5p might be promising targets used by TFDM to exert therapeutic effects and synergistic action in the treatment of VaD.

In this study, 3,522 aberrantly expressed mRNAs were identified based on VaD microarray analysis, including 1,478 upregulated and 2,044 downregulated mRNAs. Among these genes with the most significant changes in expression, corticotropin-releasing hormone (CRH) and CD163 have a previously confirmed correlation with the occurrence and pathology of VaD (Heilig et al., 1995; Guo et al., 2018; Landis et al., 2018; Short et al., 2019). Several significantly upregulated genes in VaD were identified as first, including Heat shock 70 kDa protein 1A (HSPA1A), V-set and immunoglobulin domain containing 4 (VSIG4), and complement C1q B chain (C1QB), all defined as vascular disease risks (Dulin et al., 2010; Bos et al., 2017; Lyu et al., 2020).

A total of 2,562 miRNAs were significantly dysregulated, divided into 1,167 upregulated and 1,395 downregulated miRNAs. Based on a comprehensive study of data mining and bioinformatics analysis, the 12 most abnormally expressed miRNAs were acquired. Among these miRNAs, miR-6784-3p, miR-6762-3p, miR-4747-3p, and miR-4746-3p were reported for the first time. Other significantly expressed miRNAs, such as miR-6777-3p, miR-3184-3p, miR-3184-5p, miR-4667-5p, and miR-6875-5p, closely implicated with the occurrence and development of a variety of tumors (Miyamoto et al., 2015; Akazawa et al., 2019; Hindle et al., 2019; Zhou et al., 2020; Liu et al., 2021), were identified for the first time in VaD, suggesting a novel potential regulatory role in the pathology of this disease. miR-3675-3p is a unique biomarker representing a target in the inhibition of cell apoptosis through the MAPK signaling pathway (Zhang et al., 2020). As a novel miRNA identified in our analysis, the downregulation of miR-3675-3p might participate in the induction of neuronal apoptosis in VaD. miR-4467 and miR-1268a showed an increased expression in VaD in the present study. The former has previously been described as a promising and robust candidate biomarker in the diagnosis of neurodegenerative diseases by its analysis in body fluids (Denk et al., 2015), while the latter is significantly associated with cerebrovascular disorders (Sonoda et al., 2019) and hypertension (Li et al., 2020), predicting the risk of cerebrovascular diseases prior to the onset of stroke.

An overlap between DEGs in these miRNA-predicted targets and the large number of DEGs in VaD was constructed to gain insight into the molecular functions of the most abnormally expressed miRNAs in VaD, then the related biological function and pathways were analyzed. GO analysis indicated that the dysregulated mRNAs performed a crucial role in transcription, post-transcription, and protein modification. The primary biological processes in which the target genes of these selected aberrant miRNAs were involved included a variety of binding processes, including the binding of nucleotides and chromatin, cell differentiation, vascular events involving blood vessel development, muscle structure development, branching morphogenesis, and extensive regulation of receptor activity. The pathway enrichment analysis indicated the involvement of RAGE, Ras/MAPK, TNF, gonadotropin-releasing hormone (GnRH), and estrogen pathways, which are known as implicated in the pathogenesis of VaD (Emanuele et al., 2005; Belkhelfa et al., 2018; Wang et al., 2019; Yang et al., 2020b). Among these enriched pathways, the RAGE signaling pathway plays a role in inflammatory reactions through the proinflammatory cytokine overproduction mediated by the nuclear factor-κB (NF-κB), such as TNF-α (Vicente Miranda and Outeiro, 2010), both in the blood-brain barrier (BBB) and in the glial responses. MAPK is also a crucial signaling pathway in the pathological process of VaD, which destroys the BBB, activates inflammatory mediators, and induces programmed cell death (Mohamed et al., 2015). Thus, these predicted biological processes and signaling pathways indicate the correlation of pathological processes within miRNA-target genes, including neuroinflammation, vascular dysfunction, altered barrier permeability, and neuronal damage.

A total of 13 central genes were obtained through the topological analysis of the PPI network. Among them, WNT5A, a Wingless-type (Wnt) family member, induces different aspects of synaptic differentiation and plasticity in the hippocampal neurons (Varela-Nallar et al., 2010). WNT5A was a predicted target of miR-4667-5p in the present work, and the protein Wnt5a that it encodes is known to induce the rapid activation of the CaMKII (Farias et al., 2009), thereby reinforcing the Wnt/Ca^2+^ signaling pathway in the hippocampal neurons. Wnt5a also evokes cortical axon outgrowth and repression by calcium signaling pathways. It is required in the Wnt5a/CaMKII signaling pathway and is essential for the binding of tau that is phosphorylated at Ser262 (Li et al., 2014). In addition, dysfunction of the Wnt signaling is linked to an increased Aβ formation, neuroinflammation, and neurotoxicity (Li et al., 2011; Tapia-Rojas et al., 2016). Therefore, a change in WNT5A expression represents a considerable potential as an indicator of VaD pathology.

Other central genes may additionally be involved in different pathological processes of neurodegeneration. For instance, AAK1, a predicted gene regulated by miR-4667-5p, encodes the adaptor-associated kinase 1 and is a modulatory enzyme of the adaptor protein complex 2 (AP-2) responsible for the regulation of clathrin-mediated endocytosis (Smythe and Ayscough, 2003). In a murine model of AD periodic variation in AAK1 expression, it is closely correlated with cognitive decline (Fu et al., 2018). AAK1 inhibitors have been further developed to provide valuable treatments for disorders such as neuropathic pain, AD, and other neurodegenerative diseases (Abdel-Magid, 2017), indicating that AAK1 serves as a suitable biomarker of cognitive disorders, including VaD. By combining the analysis of various functionalities, the selected miRNAs and their central target genes were screened as biomarkers or potential targets for direct or indirect involvement in VaD.

Notably, the present study indicates that TFDM has multiple targets and influences numerous pathways that prevent and treat VaD. On the one hand, TFDM contains 42 flavonoids, reflecting the multi-component action. On the other hand, a single flavonoid component had multiple targets. Thus, a molecular network was established based on the target prediction of each flavonoid contained in TFDM using Cytoscape, which revealed 43 key genes related by TFDM against the pathophysiology of VaD. Among the flavonoids of TFDM, common neuroprotective compounds such as kaempferol, chrysoeriol, apigenin, luteolin, quercetin, and salvigenin exhibited a favorable target docking score performance. Furthermore, eigenvector centrality was used to express the importance of the nodes; those present in a free state (aglycones) exhibited much stronger potential activity toward the targets than the sugar-bound flavone glycosides.

Kaempferol, one of the active compounds in TFDM, is also an essential ingredient in the extracts of *Ginkgo biloba* leaves, having antioxidant properties by acting as a free radical scavenger (Ahlemeyer and Krieglstein, 2003). Kaempferol plays a role in the regulation of inflammation and apoptosis through the targeting of the PI3-kinase p110-gamma subunit (PIK3CG) (Shen et al., 2020). Luteolin, another active ingredient of TFDM, displays neuroprotective effects on the BBB by inhibiting fibrillary Aβ_1–40_-induced inflammation through the inhibition of the p38 MAPK signaling pathways, as reported by our previous work (Zhang et al., 2017). Quercetin, also reported by our research group, provides neurovascular protection by reducing oxidative damage, inactivating RAGE-mediated pathways, and maintaining cholinergic neuron function, thereby providing an alternative medication for neuroprotection (Liu et al., 2013). Tilianin, an indicative reference of TFDM, ameliorates the cognitive dysfunction and neuronal damage in VaD by modulating p-CaMKII-mediated long-term memory, oxidation and inflammation associated with the oxidized-CaMKII (Jiang et al., 2021). Taken together, these active compounds might be considered ingredients effective in the treatment of VaD. Furthermore, the underlying mechanisms and potential targets of these active ingredients in TFDM were located into the purpose of the current bioinformatics analysis. This might support the TFDM-mediated neuroprotection on the attenuation of the Aβ-induced cognitive decline and cardiocerebrovascular deficit by restoring the anti-amyloidogenic, neurotrophic, and MAPK pathways (Liu et al., 2018; Zeng et al., 2018).

The interactions between the targets and potential binding of miRNAs were analyzed to further explore the potential effective molecules and investigate the underlying mechanism used by these active compounds in TFDM. As demonstrated in the present study, miR-3184-3p might be a potential target gene of all the compounds in TFDM against VaD. miR-3184-3p targeting the PIK3CG was regulated by apigenin, luteolin, and quercetin against VaD. Another most enriched target miRNA was miR-6875-5p that could be regulated by 37 active components of TFDM. miR-6875-5p/G protein-coupled receptor kinase 6 (GRK6) and miR-6875-5p/RPS6KA3 might be responsible for the beneficial action of apigenin, luteolin, and tilianin. According to the results of this study, highly compound-enriched miR-6777-3p was targeted by apigenin, luteolin, quercetin, and kaempferol in the protection of blood vessels by regulating the expression of the steroid hormone receptor ERR1 (ESRRA) and CAMK2. In addition, other axes, including miR-3184-5p/ornithine decarboxylase (ODC1), miR-3675-3p/BDNF/NT-3 growth factors receptor (NTRK2), miR-4667-5p/Protein kinase C alpha type (PRKCA), miR-6784-3p/Lysosomal acid glucosylceramidase (GBA), and miR-6875-5p/MAPK14, are the most likely key mediators in the pathogenesis of VaD. Therefore, the discovery of novel miRNAs that mediate specific signaling pathways provides new insight into the function and molecular mechanisms of TFDM, which acts as a candidate for a multiple targeting therapy.

The expression of 5 miRNAs that were most closely related to TFDM in the treatment of ischemic injury was measured by qRT-PCR. The expression of miR-3184-3p and miR-6875-5p in OGD-injured neuronal cells matched the bioinformatics results, suggesting that both miRNAs might play essential roles in the VaD process. In line with the neuroprotective effects of TFDM with respect to OGD injury, the abnormal expression of miR-3184-3p and miR-6875-5p was restored by TFDM, as well as by the active compounds tilianin, luteolin, and apigenin.

Although the present study described putative new biomarkers for VaD, identifying a number of promising DEMs, the results require a greater depth of experimental confirmation, such as the function and interaction assays of key genes *in vitro* and *in vivo*. Based on bioinformatic analysis and experimental verification, miRNA-mediated RAGE signaling and CaMKII-induced multi-target pathways should be confirmed, as well as the underlying molecular basis of key genes and miRNA molecules in turn to confirm the therapeutic effects of TFDM on VaD.

## Conclusion

In conclusion, differentially expressed miRNAs, mRNAs, and their functional properties during the pathogenesis of VaD were identified as contributors through bioinformatics analysis, thus revealing the therapeutic efficacy and molecular mechanisms of TFDM. The qRT-PCR validation suggested that miR-3184-3p and miR-6875-5p might be potential targets of TFDM, which might mediate multiple signaling pathways involved in the pathology of VaD.

## Data Availability

The datasets presented in this study can be found in online repositories. The names of the repository/repositories and accession number(s) can be found in the article/Supplementary Material.
